# Cellular Distribution of Brain Aquaporins and Their Contribution to Cerebrospinal Fluid Homeostasis and Hydrocephalus

**DOI:** 10.3390/biom12040530

**Published:** 2022-03-31

**Authors:** José Luis Trillo-Contreras, Reposo Ramírez-Lorca, Javier Villadiego, Miriam Echevarría

**Affiliations:** 1 Institute of Biomedicine of Seville (IBiS), Virgen del Rocío University Hospital, (HUVR)/Spanish National Research Council (CSIC)/University of Seville, 41013 Seville, Spain; josetricon@gmail.com (J.L.T.-C.); reporamirez@us.es (R.R.-L.); 2 Department of Physiology and Biophysics, University of Seville, 41009 Seville, Spain; 3 Network Center for Biomedical Research in Neurodegenerative Diseases (CIBERNED), 28031 Madrid, Spain

**Keywords:** aquaporins, cerebrospinal fluid, choroid plexus, ependyma, single-cell RNA, sequencing, hydrocephalus, aging, hypoxia

## Abstract

Brain aquaporins facilitate the movement of water between the four water compartments: blood, cerebrospinal fluid, interstitial fluid, and intracellular fluid. This work analyzes the expression of the four most abundant aquaporins (AQPs) (AQP1, AQP4, AQP9, and AQP11) in the brains of mice and discuss their contribution to hydrocephalus. We analyzed available data from single-cell RNA sequencing of the central nervous system of mice to describe the expression of aquaporins and compare their distribution with that based on qPCR, western blot, and immunohistochemistry assays. Expression of AQP1 in the apical cell membrane of choroid plexus epithelial cells and of AQP4 in ependymal cells, glia limitans, and astrocyte processes in the pericapillary end foot is consistent with the involvement of both proteins in cerebrospinal fluid homeostasis. The expression of both aquaporins compensates for experimentally induced hydrocephalus in the animals. Recent data demonstrate that hypoxia in aged animals alters AQP4 expression in the choroidal plexus and cortex, increasing the ventricle size and intraventricular pressure. Cerebral distensibility is reduced in parallel with a reduction in cerebrospinal fluid drainage and cognitive deterioration. We propose that aged mice chronically exposed to hypoxia represent an excellent experimental model for studying the pathophysiological characteristics of idiopathic normal pressure hydrocephalus and roles for AQPs in such disease.

## 1. Introduction

Water is the main compound of all living organisms and is essential for life. All organic ions and most macromolecules that form the cells are dissolved in water, and water is involved in most biological processes, such as homeostasis and the osmotic balance of cells. Little more than 30 years ago, hypotheses were debated and proposed as to the mechanism by which water crosses biological membranes [1,2,3]. The molecule’s small size, absence of electrical charge, low solubility in the membrane’s lipid phase, and its low activation energy to permeate in certain cells led to the consideration of the presence of preferential pathways for the passage of water across the plasma membrane [2,3,4,5,6]. In contrast, the presence of certain membranes with very low water permeability, such as in the renal cells of the ascending segment of the loop of Henle, showed that water permeability is not a constitutive property of the lipid bilayer [7]. The finding that the movement of water through the membranes of red blood cells could be blocked by mercury compounds derivatives [2,8,9] suggested the presence of specialized proteins in the formation of aqueous pores in the plasma membrane, whose expression and function could vary in different cell types. It was in such a scenario that Preston and Agre (1992) found a 28 kDa polypeptide, whose structural analysis revealed it as a new integral membrane protein not related to the rhesus (Rh) group of blood proteins [10]. This protein was called the channel-forming integral protein of 28 kDa (CHIP-28) and would later receive the name aquaporin 1 (AQP1) [11,12]. Since then, more than 450 different varieties of aquaporins have been identified, being present in all kingdoms of nature, from single-cell organisms such as archaea, bacteria, and yeast to multicellular organisms such as fungi, plants, and animals, with multiple genes that express different aquaporins, demonstrating the basic role of these proteins in maintaining the physiological functions of all organisms [13,14,15].

**Figure 1:**
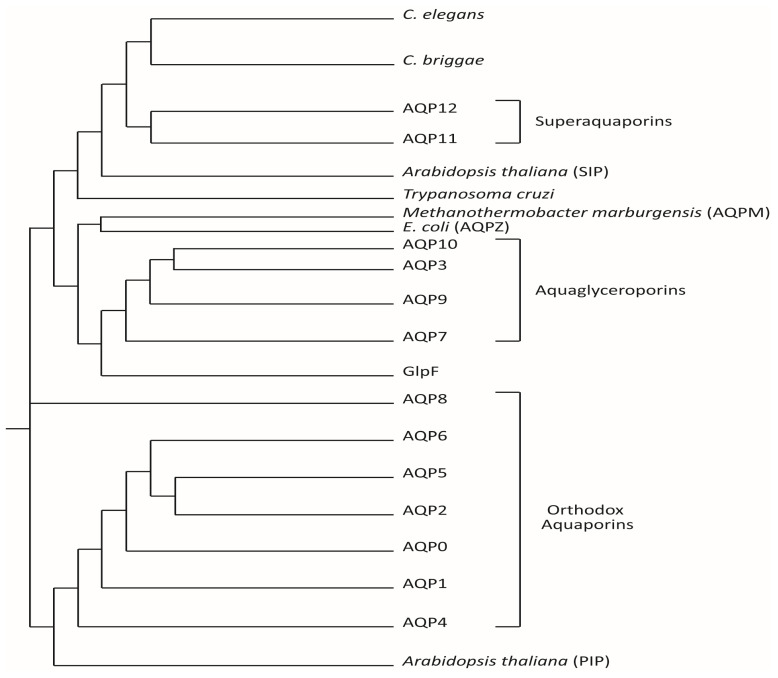
Phylogenetic tree of human aquaporins and other known aquaporins. The 13 aquaporins known to date in humans are represented, according to the homology in their amino acid sequence for each member of the family, differentiating between “strict” aquaporins (water aquaporins, at the bottom) and aquaglyceroporins (at the top). Superaquaporins, the last aquaporins identified in mammals are also shown and are the most distant in their amino acid sequence homology from the two groups mentioned above but are closer to the aquaporins expressed in species of intracellular parasites such as *Trypanosoma cruzi* (TcAQP) and of nematodes such as *Caenorhabditis elegans* and *Caenorhabditis briggsae*. The tree also shows certain bacterial aquaporins such as GlpF, a facilitator of the passage of glycerol, which are similar in their sequence to the aquaglyceroporins, and AQPZ from *E. coli*, as well as the aquaporin from methanogen archaea, AQPM from *Methanothermobacter marburgensis* and the plant aquaporins from *Arabidopsis thaliana* (small basic intrinsic protein [SIP] and plasma membrane intrinsic protein [PIP], respectively). Modified from Gorelick et al., 2006 [17].

In humans, up to 13 different aquaporins have been cloned and characterized based on their homology to the sequence of the major intrinsic protein family (Figure 1). Based on their structure and permeability, three subfamilies of aquaporins are currently differentiated in mammals: (1) the classical or orthodox aquaporins (AQP0, AQP1, AQP2, AQP4, AQP5, AQP6, and AQP8), which act as canonical water channels; (2) the aquaglyceroporins (AQP3, AQP7, AQP9, and AQP10) that are permeable to water, glycerol, and certain small solutes with neutral charge; and (3) the superaquaporins (AQP11 and AQP12), which are the subfamily with an amino acid sequence most distant to the rest of the major intrinsic protein members and are water-permeable; AQP11 can also facilitate the diffusion of glycerol and hydrogen peroxide through the membrane [16,17,18]. Recently, other AQPs besides AQP11 have been described as permeable to hydrogen peroxide (AQP1, AQP3, AQP5, AQP8, AQP9), and constitute the so called peroxiporins [19].

## 2. Expression in the Cell Membrane

Aquaporins are small proteins whose size ranges from 255 to 342 amino acids (approximately 30 kDa) and consist of six α-helices, joined by five connecting loops, with the terminal amino and carboxyl domains located on the intracellular side. The water permeability of aquaporins is very high: up to 3 × 10^9^ water molecules are estimated to pass through each monomer per second [16,20]. Since they were discovered, aquaporins have attracted attention not only for their significant water transport capacity but also for their high selectivity and tetrameric arrangement in the cell membrane. Each monomer of an aquaporin constitutes an independent pore. Electronic crystallography and X-rays made it possible to determine that these monomers are assembled into a homotetrameric structure in the membrane [21]. The four monomers form a central pore whose function has not yet been clearly established [22,23].

Aquaporin-4 (AQP4) is the only known aquaporin to show a higher ordering level in the membrane. Tetramers are assembled in supramolecular structures called orthogonal arrays of particles (OAP) [24], which are stabilized by the interaction between aminoterminal residues of AQP4 monomers. AQP4 presents various isoforms that are generated by alternative splicing [25,26], the two best known of which are AQP4-M1 and AQP4-M23. The two isoforms differ by 22 amino acids, due to the presence of two translation initiation sites, one in methionine Met1 (AQP4-M1) and the other in Met23 (AQP4-M23) [27]. The shorter N-terminal end allows the formation of orthogonal structures or matrices of the isoform homotetramers AQP4-M23. Although the longer and voluminous N-terminal of AQP4-M1 hinders the stabilization of the crystalline structure due to steric obstacles [28,29], no differences in the water permeability of biological membranes have been detected for either of these isoforms [30]. The only differences in biological function among both isoforms have been observed regarding the capacity that AQP4-M23 has to form OAP in the membrane respect to AQP4-M1 that seems to be less efficient forming those structural arrays. Although the function of OAPs is still unknown, it has been speculated that alterations in this organization could contribute to explain certain pathologies, as neuromyelitis optica. In this autoimmune disease, the presence of serum IgG autoantibodies against AQP4 is an effective marker for the accurate diagnosis of the disease [31]. The AQP4-M23 isoform appears to activate the immune system, in fact, neuromyelitis optica autoantibodies show greater reactivity against AQP4-M23 than AQP4-M1 [32]. Moreover, it has also been suggested that differences in the M1-M23 ratio might lead to changes in cell migration and adhesion in astrocytes [27].

## 3. Aquaporin Expression in the Central Nervous System

Aquaporin expression is not restricted to a single organ or tissue (Table 1); aquaporins present in the central nervous system are therefore not exclusive to it and can be found elsewhere.

**Figure 2:**
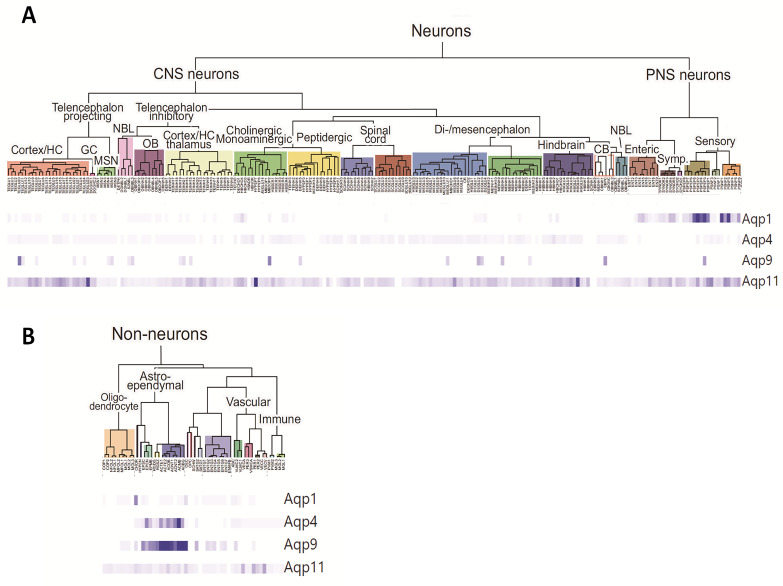
Molecular study of the mouse nervous system using single-cell RNA sequencing technology. The 265 distinct cell clusters identified based on the analysis of 160,796 transcriptomes of the mouse nervous system: brain, spinal cord, dorsal and peripheral sympathetic ganglia, and enteric nervous system are represented and classified in the tree diagrams; (**A**) Tree diagram for Neuron clusters, and (**B**) Tree diagram for non-neuron clusters. The represented dendrogram has been constructed using the digital resource (http://mousebrain.org; accessed on 23 October 2019); prepared in the laboratory of Sten Linnarsson, obtained from Zeisel et al. (2018) [81], and shows the relationship between cell types and the gene expression of AQP1, AQP4, AQP9, and AQP11. The color-graded bands at the bottom of the figure show the expression pattern of each gene in the 265 identified cell types. Each band represents the relative expression of a transcript in a cell type with respect to the maximum expression detected for that same transcript in any cell type. Given that they are relative data, the richness of expression of the different transcripts cannot be compared with each other, but they do help locate where the expression occurs. Thus, AQP4 and AQP9 appear to show a very similar expression pattern, clearly predominant in the astroependymal line, although their expression levels are very different. AQP1 expression is well defined in the dorsal ganglia and ependymal cells of the choroid plexus and contrasts with the high delocalization of AQP11. The data on absolute expression are represented in Table 2.

In the brain, AQPs mediate the movement of water between the different fluid compartments (intracellular fluid, interstitial fluid [ISF], cerebrospinal fluid [CSF], and blood) [62,63,64,65,66]. Currently, 9 AQPs have been identified in different sites in the central nervous system (CNS): AQP1 [67,68,69], AQP3 [70,71], AQP4 [72,73,74], AQP5 [75], AQP6 [76,77], AQP7 [71,74,78], AQP8 [53,70,79,80], AQP9 [30,31,71,74], and AQP11 [17,58] (Figure 1 and Figure 2). Table 1 describes the location and certain properties of the aquaporins expressed in the CNS, as well as a number of conditions associated with their dysfunction in humans.

Using recently available data generated from single-cell RNA sequencing technology in the CNS of mice [81], we analyzed the expression of the three most widely expressed brain AQPs, namely AQP1, AQP4, and AQP9. The first two (AQP1 and AQP4) are aquaporins strictly permeable to water and extensively associated with CSF homeostasis [63,64,65,66], while AQP9, an aquaglyceroporin, might play a role in brain energy metabolism [63,64,65,66]. Data on AQP11, the most recent aquaporin detected in the brain, were also included in the analysis [17,58] (Table 1), allowing us to compare the findings based on classical reverse transcription polymerase chain reaction (RT-PCR), western blot, and immunostaining, with this novel and powerful tool for detecting gene expression at the unique cell level. The transcriptome analysis performed by Zeisel et al. [81] of half a million single cells obtained from the mouse nervous system helped identify 265 clusters of distinct cell types and determine the specific repertoire of genes expressed in them. The classification of each cell cluster was organized based on three aspects: (1) cell type (e.g., neurons, astrocytes), (2) developmental/anatomical region (e.g., telencephalon, midbrain, diencephalon), and (3) neurotransmitter type (e.g., dopamine, gamma aminobutyric acid, glutamate). Lastly, the gene expression pattern for each cell cluster was obtained based on the expression frequency of a given gene in each cell of a cell cluster with respect to the expression in all the analyzed cells.

Expression levels for AQP1, AQP4, AQP9, and AQP11 in each of the 265 identified cell clusters were obtained by using the tool provided by Zeisel et al. (http://mousebrain.org; accessed on 23 October 2019) (Figure 2, Table 2). As shown in Table 2, the data analyzed by single-cell RNA sequencing validate the high AQP1 expression in cell cluster #224, corresponding to choroid plexus epithelial cells (#224-CP epithelial cells: 1.93), thereby corroborating the AQP1 expression previously reported in the plasma membrane of epithelial cells of the choroid plexus in the brain of primates and rodents. A number of studies have also identified the presence of AQP1 in the dorsal horn of the spinal cord and in the sensory ganglion of the trigeminal nerve associated with nociception [73]. In fact, the single-cell RNA sequencing analysis shows abundant AQP1 expression in cell groups #200–214, representative of the dorsal root ganglia of the spinal cord (Table 2). Moreover, single-cell RNA sequencing data supported the presence of AQP1 in the afferent pathway of the fifth cranial nerve, of the trigeminal nerve (#69 -Afferent nuclei of cranial nerves III–V: 0.39), as well as in the ganglia of motor neurons (clusters# 194, 195: 0.81–0.84).

AQP4, the most abundant aquaporin in the CNS, is mainly located in the ependymal-glial-limiting membranes, including the processes of the subpial astrocytes in the cortex, the subependymal glia and the ependymal cells that border the brain ventricles, and in the feet of the perivascular astrocytes that surround the blood vessels forming the blood-brain barrier [53,74]. This AQP4 distribution has been corroborated with the expression data by single-cell RNA sequencing (Table 2). We observed that AQP4 presents a highly marked expression pattern in ependymal cells (cluster #227-Ependymal cells: 3.61; cluster #228-Ependymal cells in the midbrain: 1.29), which are cylindrical-shaped ciliated cells bordering the brain ventricles that participate in CSF circulation and homeostasis. AQP4 is also highly expressed in subventricular zone radial glia-like cells (cluster #230: 0.43) and in various astrocytes (clusters #231–236: 1.09–10.5).

In general, the data reflected in Table 2 confirm that AQP4 is the cerebral aquaporin with the broadest and highest expression in the mouse CNS. Although AQP4 is also detected at extremely low levels in neurons, its expression is mainly localized in ependymal cells and astrocytes, with significant differences in expression levels between various types of astrocytes. For example, there is a relative abundance of AQP4 expression in fibrous astrocytes in the cerebral cortex compared with protoplasmic cortical astrocytes (cluster #231—Telencephalon astrocytes, fibrous: 2.94; vs. cluster #232—Telencephalon astrocytes, protoplasmic: 1.09). Additionally, differential AQP4 expression can be observed in fibrous versus protoplasmic astrocytes from non-tencephalic regions, such as those of the thalamus, medulla oblongata, and spinal cord (cluster #235—Non-telencephalon astrocytes, fibrous: 3.80; vs. cluster #234—Non-telencephalon astrocytes, protoplasmic: 2.10). Notable expression was observed in other glial cells, such as astrocytes in the olfactory bulb and Bergmann glia (cluster #233—Olfactory astrocytes: 3.25; cluster #237—Bergmann glia: 2.49). Interestingly, the cell type with the highest AQP4 expression is a specific cell cluster of astrocytes located in the dorsal midbrain, with relative expression levels above 10 (cluster #236—Dorsal midbrain Myoc-expressing astrocyte-like: 10.5). Consistent with this individual cell RNA expression, previous findings have already reported AQP4 expression in sensory organs such as Müller cells in the retina [82], in the olfactory epithelium, and in Claudius and Hensen cells in the inner ear [82,83].

AQP9 expression in the CNS has been reported in astrocytes, ependymocytes, tanycytes (specific ependymocytes in the third and fourth ventricles that send processes to the hypothalamus), endothelial cells of pial vessels, and dopaminergic neurons of the midbrain (ventral tegmental area and substantia nigra) [84]. The similarities in expression patterns between AQP4 and AQP9 in the brain has led to the proposal that both proteins could act synergistically in the transfer of water between the CSF and brain parenchyma. An analysis of the expression data by single-cell RNA sequencing effectively reflects that AQP9 is predominantly distributed in the astroependymal line. However, AQP9 expression is much lower than that observed for AQP4, being below the threshold value (0.2), which considers that the gene expression is positive in a group of cells (Table 2). Therefore, AQP9 expression, although very minor, was found in the dopaminergic neurons of the ventral tegmental area and the substantia nigra (cluster #77—Dopaminergic neurons: Substantia nigra and Ventral tegmental area: 0.0146) and in the same groups (clusters #227–237: 0.01–0.12) of the astroependymal type, where the AQP4 expression was indicated above.

Of the various aquaporins identified in the CNS, AQP11 is the least known and has been proposed to play an important role in the cellular response to oxidative stress, as well as in the translation and protein-folding processes, given its location in the endoplasmic reticulum [19,58,85]. The presence of AQP11 has been reported in the cerebellum, specifically in the dendrites of Purkinje cells, in neurons from the CA1 and CA3 regions of the hippocampus, in neurons from the cerebral cortex (layers II–VI), and in endothelial cells from the brain parenchyma and choroidal plexus epithelial cells [17,58]. The single-cell RNA sequencing analysis revealed the broad AQP11 distribution in almost all cell clusters identified in this study, although AQP11 expression is very minor and in many cases below the threshold. Specifically, nonsignificant AQP11 expression was observed in the neurons in the cerebellum, in Purkinje cells (clusters #172–178: <0.2; data not shown), and in choroidal plexus epithelial cells (cluster #224—Choroid plexus epithelial cells: 0.07). In other regions such as the hippocampus (cluster #21—Excitatory neurons, hippocampus CA1: 0.22; cluster #24—Excitatory neurons, hippocampus CA3: 0.77; cluster #57—Trilaminar cells, hippocampus: 0.22), cerebral cortex (clusters #7–17: Excitatory neurons, Cerebral Cortex: >0.2), and vascular endothelial cells (cluster #254—Vascular endothelial cells, arterial: 0.21; cluster #257—Pericytes: 0.23; cluster #260—Vascular endothelial cells, venous: 0.29), the data show a greater presence of AQP11, validating previous reports. Lastly, it is worth highlighting the strong expression observed in hypothalamic neurons (cluster #73—Pmch neurons Hypothalamus: 1.23), which could be associated with the endocrine function.

## 4. The Role of Aquaporins in Cerebrospinal Fluid Homeostasis

Since the beginning of the twentieth century, physiologists interested in understanding CSF homeostasis have accepted that the circulation of cerebrospinal liquid has a unidirectional path [86,87]. Consequently, with the “classic view”, CSF would be produced primarily by the choroid plexus to fill the ventricles and circulate unidirectionally toward the subarachnoid space and the central canal of the spinal cord, ultimately draining through arachnoid granulations into the meningeal sinus or through lymphatic vessels of the nasal mucosa after crossing the cribriform plate [85,88]. Regarding the role of aquaporins in this circuit, AQP1 was classically associated with CSF production, given its exclusive presence in the apical membrane of choroidal plexus epithelial cells. AQP4, abundantly present in ependymal cells, glia limitans, and astrocyte processes in the pericapillary end foot, was mainly associated with ISF/CSF exchange and CSF absorption. However, several findings led to an updating of the classic concept to a more complex hypothesis (but one surely closer to reality) for understanding CSF production and circulation [89]. Experiments with aquaporin knock-out animals have definitively contributed to clarifying the role of aquaporins in the CSF.

In 2005, the study by Oshio et al. [90] using AQP1^−/−^ mice essentially demonstrated the prominent role of AQP1 in CSF formation. In these knock-out animals, a decrease in CSF production of approximately 20% and reduced intraventricular pressure (IVP) demonstrated the participation of AQP1 in CSF production. However, experiments in which ^17^O-labeled water (H_2_O^17^) was administered intravenously in AQP1^−/−^ and AQP4^−/−^ mice showed that water entry into the lateral ventricles was significantly reduced in AQP4^−/−^ mice but not in AQP1^−/−^ mice, indicating the important role of AQP4 in CSF formation [91]. Concomitantly, the finding also supports the important and constant formation of large amounts of CSF distributed in the brain as a consequence of water exchange between the brain capillaries and ISF [92], and between ISF and CSF through ependymal cells. Furthermore, the description of AQP4-facilitated perivascular flow regulating the CSF/ISF exchange between periarterial and perivenous Virchow-Robin spaces [93,94,95] that could respond to a so-called “glymphatic” system for the drainage of CSF and proteins [93] provided additional support for the important role of AQP4 in CSF homeostasis.

Using single AQP1^−/−^, AQP4^−/−^, and double-AQP1^−/−^:AQP4^−/−^ knock-out mice, our recent study strongly reinforced the clear role of both aquaporins in CSF homeostasis. When we evaluated the ventricular volume by magnetic resonance imaging, as a readout of CSF formation, we confirmed that CSF production is reduced by the lack of not only AQP1 but also AQP4 and to a similar degree [96]. The results confirm that both choroidal-expressed AQP1 and extrachoroidal-expressed AQP4 contribute comparably to CSF production and IVP. Furthermore, both aquaporins also play a role in the CSF evacuation process and in ventricular compliance [96]. We found that although the absence of either of the two AQPs did not significantly affect CSF output at low perfusion values, the simultaneous lack of both proteins significantly reduced CSF efflux and distensibility of the cerebral ventricular system. However, it is still unclear how the lack of these cerebral aquaporins could modify the structural properties of the CSF drainage routes, the ventricle ependyma, or the cerebral parenchyma so as to modify these processes.

In addition to AQP1 and AQP4, other aquaporins are also expressed in the brain, as previously indicated. Of these, AQP9 and AQP11 appear to be the next most abundant, but their specific contribution to the various aspects of CSF homeostasis and dynamics is still unknown. As indicated in the previous section, AQP9 is localized in astrocytes, ependymal cells, and a few neuronal groups (Table 2), a distribution very similar to that of AQP4. However, the low AQP9 expression found in these cell types makes it difficult to propose a functional role for this protein in relation to CSF homeostasis. In contrast and given the significant vascular AQP11 expression in pericytes and arterial and venous endothelial cells (Table 2), it could be hypothesized that AQP11 is expressed in endothelial cells and pericytes synergizes with the AQP4 located in the astrocytic endfeet to facilitate the CSF/ISF exchange between the periarterial and perivenous Virchow-Robin spaces, thereby contributing to both CSF production and clearance.

## 5. Aquaporin Expression Is Modified in Experimental Models of Hydrocephalus

Hydrocephalus is a pathological condition characterized by CSF accumulation in the cerebral ventricles and subarachnoid space, typically leading to increased intraventricular pressure (IVP). Hydrocephalus is the consequence of an imbalance between CSF formation and drainage and of circulation abnormalities [85,97,98] resulting from numerous reasons, such as genetic origin, malformation, intracranial hemorrhage, brain tumors, infections, and chronic conditions such as idiopathic normal pressure hydrocephalus (iNPH). In addition, changes in brain aquaporin expression have recently been associated with hydrocephalus, and various experimental models have been employed to study their contribution to this condition. The injection of kaolin into the cisterna magna has been widely used in rodents to produce hydrocephalus; in such an experimental model, AQP1 and AQP4 levels have been extensively explored. An increase in AQP4 mRNA was thereby observed in the hippocampus and parietal cortex of rats after a kaolin injection [99]. Similarly, a higher AQP4 expression was observed in the blood-ISF-CSF interfaces in another model of secondary communicating hydrocephalus, due to inflammation of the arachnoid membranes by the intraparenchymal injection of stearoyl L-α-lysophosphatidylcholine [100,101]. These findings suggest an adaptive role for AQP4 in resolving the hydrocephalic condition [102]. Furthermore, the studies by Bloch et al. showed that kaolin-induced hydrocephalus progressed more rapidly in AQP4^−/−^ mice than in wild-type (wt) animals, with a higher degree of ventriculomegaly and elevated IVP [103]. Regarding the role of AQP1 in kaolin-induced hydrocephalic rats, a decrease in AQP1 mRNA levels has been observed [104], and studies in mice have reported significant AQP1 redistribution in the form of intracellular vesicles of the choroidal epithelium. The authors attribute these changes to a compensatory mechanism in decreasing CSF production and reducing the increase in IVP. AQP1^−/−^ mice were found to have a lower degree of ventricular dilation after the kaolin injection than the wt controls, supporting the participation of AQP1 in the pathophysiology of hydrocephalus [105].

All of these changes demonstrate that aquaporins respond accordingly, as a compensatory process, to prevent hydrocephalus. However, changes in aquaporin expression might contribute to hydrocephalus. It would therefore make sense that aquaporin overexpression in the choroid plexus would result in greater CSF production; in contrast, down-regulation of AQP4 expression in ependymal cells, glia limitans, and pericapillary astrocyte endfoot processes would in turn diminish the CSF exchange or evacuation, thereby contributing to hydrocephalus.

## 6. Abnormal Aquaporin Expression Might Be Associated with the Origin of Hydrocephalus

Recent studies, including ours, have shown the relationship between changes in aquaporin expression or distribution associated with ischemia, hypoxia, or aging and the promotion of a hydrocephalic state. Ischemia has generally been associated with edema more than with hydrocephalus. When a cerebral infarction or ischemic stroke occurs, edema and a concomitant increase in intracranial pressure (ICP) are the primary complications that need resolving [106]. Permanent blocking of the cerebral blood flow by two-vessel occlusion or by sealing the middle cerebral artery showed edema of the choroid plexus after 24 h [107]. However, the discrepancies between the massive increase in ICP and the volume of edema suggest that ischemic stroke can increase CSF production, contributing to the increased ICP. An ischemic stroke is a major insult to the CNS and can profoundly modify the functioning of cells and tissues involved in CSF production such as the choroid plexus, which in turn might respond by increasing CSF secretion and subsequently increasing ICP.

To date, no study has shown changes in aquaporin expression in the choroid plexus in response to ischemia. On the contrary, changes in the perivascular endfeet expression of AQP4 in response to stroke are associated with the movement of water and the presence of edema [108]. A dual role for AQP4 in edema has been proposed: contributing to the formation of edema [109] and facilitating water clearance during the resolution of this pathophysiological condition [110,111]. It is well known that, in the perivascular astrocyte endfeet cell membrane, AQP4 forms part of a complex of proteins (with syntrophin, dystrophin-dystroglycan, laminin, and agrin), which serve to anchor the astrocyte to extracellular elements, facilitating water movement in the perivascular space [112,113]. However, very little is known about the molecular effects of ischemia on astrocyte endfeet AQP4 expression. In a recent study, Filchenko et al. [114] showed that the perivascular presence of AQP4 certainly correlates with caveolin-1 expression. The lack of caveolin-1, in Cav-1^−/−^ knockout mice resulted in decreased AQP4 expression and impaired perivascular AQP4 coverage after cerebral ischemia, which in turn is associated with increased brain swelling in this mouse type. This relationship between caveolin-1 and AQP4, shown in the context of ischemia, indicates that there might be mechanisms that modify the water permeability of the perivascular barrier, based on the AQP4 intracellular relocation, in response to certain stimuli.

In general, AQP4 expression in the choroid plexus is not a widely observed phenomenon; however, various authors, including ourselves, have indicated a certain level of AQP4 expression in this location [115]. In situ hybridization and western blot analysis [116,117] has shown the presence of AQP4 in choroidal plexus cells, and our recent results also confirm (using various strategies including AQP4 immunofluorescence) AQP4 expression in the secretory epithelium [96,115]. In the human choroid plexus, recent studies by Deffner et al. [118] confirmed the presence of AQP4 expression in samples obtained from elder donors. The authors hypothesized that AQP4 expression in choroid plexus cells was caused by age-related changes. In terms of changes in the mice’s AQP4 expression, the same study confirmed that AQP4 mRNA levels were higher in the aged animals than in the adult and younger ones. The AQP4 signal in immunohistological studies often shows a diffuse cytosolic distribution [115,117] that contrasts with the clear membrane staining obtained with AQP1. Therefore, the specific subcellular location of AQP4 in epithelial choroidal cells needs to be fully explored. AQP1 located in the apical membrane of choroidal cells is the main route for the exit of water towards the ventricle. If there is also a route mediate by aquaporins for the entry of water into the basolateral membrane of this cells, however, it is still unknown, but AQP4 could represent a good candidate for this function. A tentative hypothesis could be that certain amounts of AQP4, stored in cytoplasmic vesicles, could be incorporated into the basolateral membrane of the choroidal cells in response to specific stimuli (e.g., hypoxia), generating a highly permeable transcellular pathway with AQP1 on the apical side and AQP4 on the basolateral membrane.

**Figure 3:**
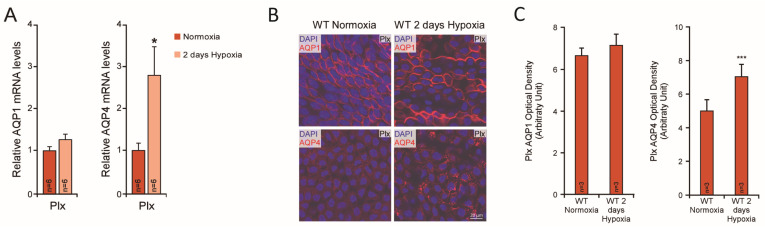
AQP1 and AQP4 expression in the choroid plexus of mice exposed to hypoxia: (**A**) The AQP mRNA expression in the choroid plexus (Plx) of mice exposed to hypoxia (10% O_2_, 48 h) and normoxic controls was analyzed by qPCR. 18S ribosomal RNA was used as the housekeeping gene for normalization (modified from Trillo et al., 2018 [115]). (**B**) AQP1 and AQP4 immunofluorescence images of the choroid plexus obtained from mice exposed to normoxia or hypoxia (10% O_2_; 48 h). AQP4 expression is notably higher after the hypoxic treatment. (**C**) Quantification by optical density of AQP1 and AQP4 expression in the choroid plexus tissue (Modified from Trillo et al., 2018 [115]) (* *p* < 0.05; *** *p* < 0.001).

Our initial study demonstrating that hypoxia increases AQP1 mRNA and protein levels in a culture of the 9L glioma cell line, through a mechanism that involves the transcription factor hypoxia-inducible factor (HIF)-1α [119], motivates us to search for the regulatory effect of hypoxia on brain aquaporins in animals. Hypoxia-induced changes in the levels of brain mouse aquaporin expression have recently been reported by Trillo et al. [96,115,120]. Contrary to our expectations, however, AQP4 and not AQP1 changed expression after hypoxic exposure. As indicated in the original study [115] (Figure 3), AQP4 mRNA and protein levels in the choroid plexus increase with exposure to hypoxia. In contrast, AQP1 expression remains unchanged by hypoxic exposure. Upregulation of AQP4 expression by HIF-1α was demonstrated in rats subjected to traumatic brain injury [121]. In primary Schwann cell cultures, HIF-1α also participates in hypoxic induction of the AQP1 gene [106]. It could therefore be possible that HIF-1α is involved in the AQP4 regulation by hypoxia reported in the choroid plexus and ependyma; however, this remains undemonstrated.

However, AQP4 expression in the cortex of hypoxia-exposed mice revealed a transient induction of the AQP4 protein that increased after two days of treatment and then decreased after five days of hypoxia. A detailed analysis showed that cortical AQP4 expression was mainly associated with astrocytes in the outer layers characterized by their high expression of glial fibrillary acid protein. In parallel with this AQP4 upregulation, an increase in ventricle size was measured, indicating mild ventriculomegaly. When this analysis was performed in aged animals, the increase in ventricular size was further enlarged in the wt and AQP1^−/−^ animals but not in the AQP4^−/−^ mice, indicating the participation of AQP4 upregulation in the hydrocephalus associated with hypoxia and aging. Recently published experimental studies (107) have demonstrated an increase in AQP4 expression with aging [118]. Several findings from our study are consistent with hypoxia-induced hydrocephalus in aged animals [115,120], similar to the main symptoms of iNPH. These findings include an increase in ventricular size, an increase in IVP, and abnormal cerebral distensibility that occurred in parallel with reduced CSF drainage and cognitive impairment. All of these changes indicate that aged mice chronically treated with hypoxia represent an excellent experimental model for studying the pathophysiological characteristics of iNPH and the therapeutic options for this disease. Growing evidence in recent years indicates that an abnormal expression or distribution of brain aquaporins could be responsible for deficient water exchange between the various fluid compartments of the brain and concomitant to the ventricle enlargement characteristic of hydrocephalus. Furthermore, the vascular and respiratory problems of sleep, as in obstructive sleep apnea, have also been associated with the etiology of iNPH. All of these pathophysiological conditions lead to intermittent events of oxygen saturation and desaturation in neural tissues, which could eventually trigger changes in the expression and distribution of brain aquaporins, inducing changes in CSF homeostasis that would eventually result in hydrocephalus. Continuing to work on this animal model and searching for correlates with findings in patients’ tissues will help advance our understanding of these diseases associated with the inadequate management of CSF.

In conclusion, the present work evidences the important role of aquaporins in the movement of water across the different fluid compartments in the brain. Brain distribution in mice of AQP1, AQP4, AQP9, and AQP11 is described here at the cellular level and the specific role of each of these proteins is indicated. The presence of AQP4 in the choroidal plexus, in addition to the widely known expression of AQP1, is outlined here, supporting a possible role for AQP4 in the production of CSF as indicated previously for AQP1. The essential role of AQP4 in the glymphatic system and in cerebral homeostasis in general is reinforced in this work and malfunction or alterations in the expression and function of brain AQP (AQP4/AQP1) has been associated with the development of chronic adult hydrocephalus or iNPH. We have recapitulated as well how synergism between aging and hypoxia could induce ventriculomegaly, reduce CSF outflow and affect cognitive function in mice in a way that recalls iNPH disease. Therefore, we propose to use this animal model (old animal/exposed to hypoxia) to further understand the contribution of AQPs to the iNPH disease and to look for proteins or other possible biomarkers that can be used to understand the origin of the disease or may help on its diagnosis.

## Figures and Tables

**Table 1:** Distribution of aquaporins in humans and the diseases associated with their dysfunction or absence. Modified from Verkman et al., 2014. [28].

Protein	Tissue Expression	Associated Diseases	Functions	References
AQP0	Fiber cells of the crystalline lens	Congenital cataracts	Cell adhesion	Verkman et al., 2002 [29]
AQP1	Erythrocytes, eye, choroid plexuses, kidneys, heart, lungs, vascular endothelium, skin, gastrointestinal tract, salivary gland, liver, testes, ovaries, and uterus, among others	Pulmonary, cerebral and peripheral edema, glaucoma, skin cancer	Urinary concentration, migration, proliferation	Ma et al., 1998 [33]; Osorio et al. [34]; Galan et al. [35,36,37].
AQP2	Renal collecting duct, ears, ductus deferens	Nephrogenic diabetes insipidus	ADH-Hormone dependent water reabsorption	Deen et al., 1994 [38]
AQP3	Kidneys, leukocytes, erythrocytes, skin, eyes, respiratory tract, gastrointestinal tract, ovaries	Skin and breast cancer	Sweating and skin elasticity. Glycerol homeostasis.	Echevarría et al., 1994; Ma et al., 2002; Levine and Verkman., 2006. [39,40,41]; Marlar et al. [42]; Osorio et al. [34]; Zhu et al. [43]; Azad et al. [44]
AQP4	Brain, retinas, salivary and lacrimal glands, olfactory and auditory epithelium, gastrointestinal tract, kidneys, lungs, and skeletal muscle	Cerebral edema, deafness, anosmia	Control of brain water balance	Manley et al., 2000; Jarius et al., 2010 [31,45]
AQP5	Epithelia of lacrimal, sweat, and salivary glands, alveolar, bronchial, and tracheal mucosa, ovaries, kidneys, and skin	Bronchial asthma, chronic bronchitis, Sjögren’s syndrome, palmoplantar keratoderma	Absorption and secretion of water in pulmonary and salivary mucosa	Tsubota et al., 2001 [46]
AQP6	Intracellular vesicles of the renal collector tubule and brain	Unidentified	Unidentified	Yasui et al., 1999 [47]
AQP7	Adipose tissue, carotid body, testes, kidneys, heart, skeletal muscle, and ovaries	Hyperglycemia, platelet defect, obesity, inflammation	Lipid metabolism from adipocytes	Skowronki et al., 2007; Muñoz-Cabello et al., 2010 [48,49]; Da Silva et al. [50]
AQP8	Liver, pancreas, gastrointestinal tract, salivary glands, kidneys, lungs and trachea, testes, and ovaries	Cancer	Bile production	Huebert et al., 2002; Saparov et al., 2007 [51,52]; Zhu et al. [53]
AQP9	Liver, leukocytes, erythrocytes, testes, ovaries, brain, kidneys, and spleen	Hyperglycemia	Hepatic lipid metabolism	Amiry-Moghaddam et al., 2005 [54]
AQP10	Small intestine, specifically in the duodenum and jejunum	Psoriasis, skin dermatitis	Lipid metabolism	Ishibashi et al., 2002 [55]; Soler et al. [56]
AQP11	Testes, heart, kidneys, ovaries, muscles, gastrointestinal tract, leukocytes, liver, brain	Obesity and Inflammation	RE stress	Morishita et al., 2005; Koike et al., 2016 [57,58]; Frühbeck et al. [59]; Ishibashi et al. [60]
AQP12	Acinar cells of the pancreas	Inflammation	Pancreatic function	Boone and Deen., 2009 [61]; Da Silva et al. [50]

**Table 2:** Gene expression pattern of brain aquaporins according to cell type analysis using single-cell RNA sequencing technology. Each row represents a cell type and the expression richness of the AQP1, AQP4, AQP9 and AQP11 genes in that cluster. The pattern is based on the calculation of the frequency of expression in a cell population, using a binomial β-Bayesian model (trinarization) of the raw data of the expression of each gene in each cell of all clusters. Therefore, the values represented are defined as the frequency of gene expression in a percentage of cells in that cluster. Values > 0.2 are considered positive, with an expression error <0.05. The data presented in the table were obtained with the digital resource (http://mousebrain.org; accesed on 23 October 2019) from Zeisel et al., 2018 [81]. #, represents a specific cell cluster, out of the 265 distinct cell clusters identified.

Index Cluster #	Name	Description Cell Types	Gene Expression			
			*Aqp1*	*Aqp4*	*Aqp9*	*Aqp11*
7	TEGLU10	Excitatory neurons, cerebral cortex	-	0.02	-	**0.26**
9	TEGLU8	Excitatory neurons, cerebral cortex	-	0.01	-	**0.27**
14	TEGLU5	Excitatory neurons, cerebral cortex	-	0.06	-	**0.21**
15	TEGLU16	Excitatory neurons, cerebral cortex	-	0.08	-	**0.26**
16	TEGLU15	Excitatory neurons, cerebral cortex	-	0.05	-	**0.35**
17	TEGLU17	Excitatory neurons, cerebral cortex	-	0.02	-	**0.27**
21	TEGLU21	Excitatory neurons, hippocampus CA1	-	0.02	-	**0.22**
24	TEGLU23	Excitatory neurons, hippocampus CA3	-	0.01	-	**0.77**
57	TEINH13	Trilaminar cells, hippocampus	-	0.02	-	**0.22**
69	HBCHO4	Afferent nuclei of cranial nerves III-V	**0.39**	0.10	-	**0.21**
73	HYPEP7	Pmch neurons, hypothalamus	-	-	-	**1.23**
77	MBDOP2	Dopaminergic neurons, ventral midbrain (SNc, VTA)	0.03	0.02	0.01	0.06
131	MEGLU6	Excitatory neurons, midbrain	-	0.05	-	**0.36**
160	HBINH2	Inhibitory neurons, hindbrain	-	**0.50**	-	**0.23**
161	HBCHO1	Cholinergic neurons, hindbrain	0.10	0.10	-	**0.20**
162	HBCHO2	Cholinergic neurons, hindbrain	-	0.05	-	**0.23**
165	HBGLU6	Excitatory neurons, hindbrain	-	0.04	-	**0.27**
167	HBGLU8	Excitatory neurons, hindbrain	-	0.09	-	**0.73**
185	ENT4	Cholinergic enteric neurons	**0.56**	0.01	-	0.01
186	ENT5	Cholinergic enteric neurons	**0.68**	-	-	0.03
188	ENT7	Cholinergic enteric neurons, VGLUT2	0.03	-	-	**0.23**
194	SYNOR4	Noradrenergic erector muscle neurons	**0.84**	-	-	**0.22**
195	SYNOR5	Noradrenergic erector muscle neurons	**0.81**	-	-	0.02
200	PSPEP6	Peptidergic (TrpM8), DRG	**0.63**	-	-	0.07
201	PSPEP5	Peptidergic (PEP1.2), DRG	**2.93**	-	-	0.10
202	PSPEP2	Peptidergic (PEP1.3), DRG	**5.70**	0.03	-	**0.28**
203	PSPEP4	Peptidergic (PEP1.1), DRG	**2.93**	-	-	0.04
204	PSPEP3	Peptidergic (PEP1.4), DRG	**4.02**	0.01	0.01	0.14
205	PSPEP1	Peptidergicv (PEP2), DRG	**2.22**	0.01	-	**0.35**
206	PSNF3	Neurofilament (NF2/3), DRG	0.03	0.03	-	**0.42**
209	PSNP1	Non-peptidergic (TH), DRG	**3.82**	0.02	-	0.07
210	PSNP2	Non-peptidergic (NP1.1), DRG	**1.49**	-	-	**0.38**
211	PSNP3	Non-peptidergic (NP1.2), DRG	**4.19**	0.01	-	**0.32**
212	PSNP4	Non-peptidergic (NP2.1), DRG	**0.47**	-	-	0.11
214	PSNP6	Non-peptidergic (NP3), DRG	**1.43**	0.03	-	**0.27**
224	CHOR	Chorid plexus epithelial cells	**1.93**	0.05	-	0.07
227	EPEN	Ependymal cells	-	**3.61**	0.01	0.03
228	EPMB	Ependymal cells, midbrain	-	**1.29**	0.01	0.04
230	RGSZ	Subventricular zone radial glia-like cells	-	**0.43**	0.02	0.01
231	ACTE1	Telencephalon astrocytes, fibrous	-	**2.94**	0.03	0.04
232	ACTE2	Telencephalon astrocytes, protoplasmic	-	**1.09**	0.04	0.04
233	ACOB	Olfactory astrocytes	-	**3.25**	0.04	0.12
234	ACNT1	Non-telencephalon astrocytes, protoplasmic	-	**2.10**	0.03	0.03
235	ACNT2	Non-telencephalon astrocytes, fibrous	-	**3.80**	0.02	0.04
236	ACMB	Dorsal midbrain Myoc-expressing astrocyte-like	-	**10.5**	0.04	0.07
237	ACBG	Bergmann glia	-	**2.49**	0.04	0.01
254	VECA	Vascular endothelial cells, arterial	-	0.02	-	**0.21**
257	PER1	Pericytes	-	0.01	-	**0.23**
260	VECV	Vascular endothelial cells, venous	-	0.01	-	**0.29**

## Data Availability

Not applicable.
